# Estimation of Gaze Detection Accuracy Using the Calibration Information-Based Fuzzy System

**DOI:** 10.3390/s16010060

**Published:** 2016-01-05

**Authors:** Su Yeong Gwon, Dongwook Jung, Weiyuan Pan, Kang Ryoung Park

**Affiliations:** Division of Electronics and Electrical Engineering, Dongguk University, 30, Pildong-ro 1-gil, Jung-gu, Seoul 100-715, Korea; gwonsuyeong@dgu.edu (S.Y.G.); jung4759@gmail.com (D.J.); westlaopan90@gmail.com (W.P.)

**Keywords:** gaze tracking, NIR camera, calibration, calibration marker and marker display, fuzzy system

## Abstract

Gaze tracking is a camera-vision based technology for identifying the location where a user is looking. In general, a calibration process is applied at the initial stage of most gaze tracking systems. This process is necessary to calibrate for the differences in the eyeballs and cornea size of the user, as well as the angle kappa, and to find the relationship between the user’s eye and screen coordinates. It is applied on the basis of the information of the user’s pupil and corneal specular reflection obtained while the user is looking at several predetermined positions on a screen. In previous studies, user calibration was performed using various types of markers and marker display methods. However, studies on estimating the accuracy of gaze detection through the results obtained during the calibration process have yet to be carried out. Therefore, we propose the method for estimating the accuracy of a final gaze tracking system with a near-infrared (NIR) camera by using a fuzzy system based on the user calibration information. Here, the accuracy of the final gaze tracking system ensures the gaze detection accuracy during the testing stage of the gaze tracking system. Experiments were performed using a total of four types of markers and three types of marker display methods. From them, it was found that the proposed method correctly estimated the accuracy of the gaze tracking regardless of the various marker and marker display types applied.

## 1. Introduction

Studies have been actively carried out to accurately calculate a user’s gaze location based on the movement of their face or eyes [1,2]. In addition, studies are being carried out to apply a user’s gaze location as a more natural and convenient input device replacing a keyboard and mouse, which are commonly used input devices [3,4,5,6,7]. Because it is intuitive, a user’s gaze location can be easily used as an input information without a separate training process. In particular, input devices such as a keyboard, mouse, or remote control, which is commonly used by the disabled who cannot use their hands freely, are difficult to apply properly. Also, even when they can be used, they cannot be conveniently manipulated or precisely controlled.

In general, gaze tracking is a technology for identifying the position on a screen that the user is looking at. In most gaze tracking systems, a user’s gaze position is calculated based on the relative positions of the center of the user’s pupil and the corneal specular reflection. Here, for an accurate gaze tracking system, it is very important to accurately find the pupil center and corneal specular reflection. Along with this, a user calibration process is necessary for the accurate gaze tracking of the user. In general, a calibration process is applied at the initial stage of most gaze tracking systems. This process is necessary to calibrate for the differences in the eyeballs and cornea size of user, as well as the angle kappa, and to find the relationship between the user’s eye and screen coordinates. It is applied on the basis of the information of user’s pupil and corneal specular reflection obtained while the user is looking at several predetermined positions on a screen. Here, if the user’s calibration process is inaccurate, it will be difficult to accurately calculate the gaze position when a gaze tracking system is executed. Therefore, to achieve a gaze tracking system with high accuracy, an accurate user calibration process is as important as the accurate detection of the user’s pupil center and reflection. In other words, if the calibration process is accurate, an accurate gaze tracking can be achieved.

Past studies in this area can be mainly divided into two types: utilizing the estimation model of a user-calibration based gaze tracking result and not utilizing such a model. The latter can be divided into cases applying and not applying a calibration process. For cases using a calibration process, the calibration methods apply static markers, dynamic markers, or a dynamic marker display. For a method not applying a calibration [8], two stereo cameras and five illuminators are applied. In this case, because no calibration process is used, user convenience increases; however, because several cameras and illuminators have to be applied, the system complexity increases, as do the price and system size.

Next, for calibration using static markers, calibration methods applying five or nine points [9], nine calibration points [10,11,12,13,14], a single position calibration [15], and four point calibration [16,17] have been utilized. In general, as the number of reference points for the calibration increases, the gaze tracking error decreases. However, as the calibration points increase in number, the user convenience decreases. In addition, because several calibration points have to be gaze focused, if a single point among several points is not focused, a decrease in the gaze tracking accuracy will occur.

For calibration using dynamic markers and a dynamic display, one such method calibrates while the user’s gaze follows a dynamically moving point [18]. In this method, a moving target appears on the screen and the calibration is conducted as the user’s gaze follows it. This method can prevent an inaccurate calibration by unexpected external disturbances. That is because the user’s concentration is high compared to the use of static markers. However, when the moving path of a calibration marker is so complex that the user cannot predict the movement, the calibration accuracy may decrease because the user’s perceptual response velocity slows down. In addition, when the movements of the marker are complex, the user must spend more time training in advance, thereby leading to a decline in user convenience. In these previous studies, a user calibration was conducted by applying various types of markers and marker display methods. However, there have been no studies conducted on estimating the accuracy of a final gaze tracking system through the results obtained from the above calibration processes. Here, the accuracy of final gaze tracking system means the gaze tracking accuracy during the testing stage of the gaze tracking system. Therefore, in this study, using a fuzzy-based estimation model based on the results obtained during the user calibration, a method for estimating the accuracy of the final gaze tracking system is proposed. Because the accuracy estimation of a gaze tracking system is made possible based on the information obtained during the calibration, a quantitative performance can be determined for various calibration markers and display methods. Compared to conventional studies, the present study is novel in the following three ways.

- The Euclidean distance is calculated between the user’s calculated gaze and the reference positions obtained during the user calibration. Then, the mean and standard deviations of the Euclidean distances are extracted as the first and second features, respectively. In addition, the change in gaze position for each frame, which is obtained during the user calibration, is extracted as the third feature.
- Developing a fuzzy system that takes these three features as inputs and the accuracy of the final gaze tracking system as an output, the accuracy of the final gaze tracking system is estimated based on the results obtained during the user calibration.
- The validity of the proposed fuzzy-based estimation system is verified experimentally using various types of markers such as static and dynamic markers, and various types of marker displays such as sequential, random, and guiding displays.

The advantages and disadvantages of this study compared to conventional studies are shown in Table 1.

**Table 1:** Comparison of the advantages/disadvantages between previous studies and the proposed method.

Categories		Method	Advantage	Disadvantage
Not using the estimation model of gaze tracking accuracy	Not conducting a user calibration	Requiring two stereo cameras and five light sources [8]	User convenience increases because of no calibration process	Due to two cameras and multiple illuminators, the system complexity increases, as do the price and system size
	Calibration using static markers	Calibration by selecting five or nine points [9]	As the number of calibration points increases, the gaze tracking accuracy becomes more accurate	Decrease in user convenience
		Nine calibration points [10,11,12,13,14]		Gaze tracking accuracy is affected by the accurate calibration procedure
		One point calibration [15]		
		Four point calibration [16,17]		
	Calibration using dynamic markers and display	Calibration by following a moving target with the eyes [18]	Prevents an inaccurate calibration because the user’s concentration is high compared to the use of static markers	When the movement line of the calibration marker is so complex that it cannot be predicted by the user, the calibration accuracy decreases
				When the movements are complex, the user convenience decreases
Using the estimation model of gaze tracking accuracy		Gaze tracking accuracy can be estimated using fuzzy system based on the user calibration information from various calibration methods using static, dynamic markers and display	The performance of gaze tracking system can be quantitatively predicted for various calibration markers and display methods	The design of an additional fuzzy rule table and the membership function is necessary
**(proposed method)**				

The remainder of this paper is organized as follows. In Section 2, the calibration-based estimation method of gaze tracking is described. In Section 3, the experimental results with various calibration methods and an analysis of these methods are provided. In Section 4, some concluding remarks and the direction for a follow-up study are given.

## 2. Proposed Method for Estimating the Accuracy of Gaze Tracking System

### 2.1. Detection of User’s Pupil Center and Corneal Specular Reflection, and Calculation of Gaze Position

**Figure 1:**
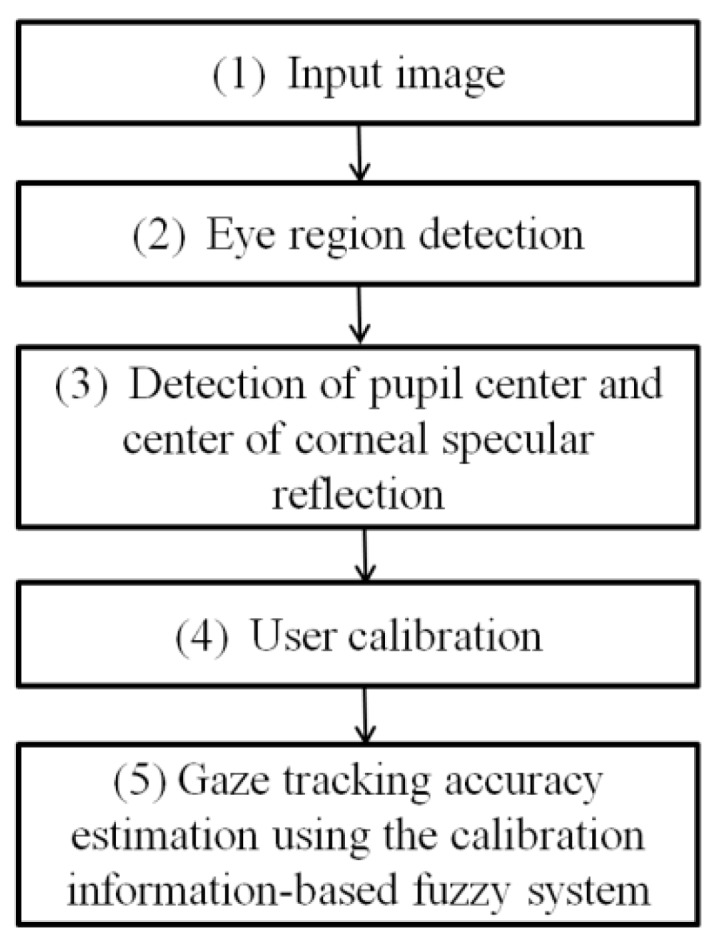
Flowchart of user calibration-information based gaze tracking estimation system.

Figure 1 shows a flowchart of the user calibration-information based gaze tracking estimation system proposed in this study. We refer to the previous research [19] for detecting the centers of pupils and corneal specular reflection.

#### 2.1.1. Pupil Center Region Detection Process

**Figure 2:**
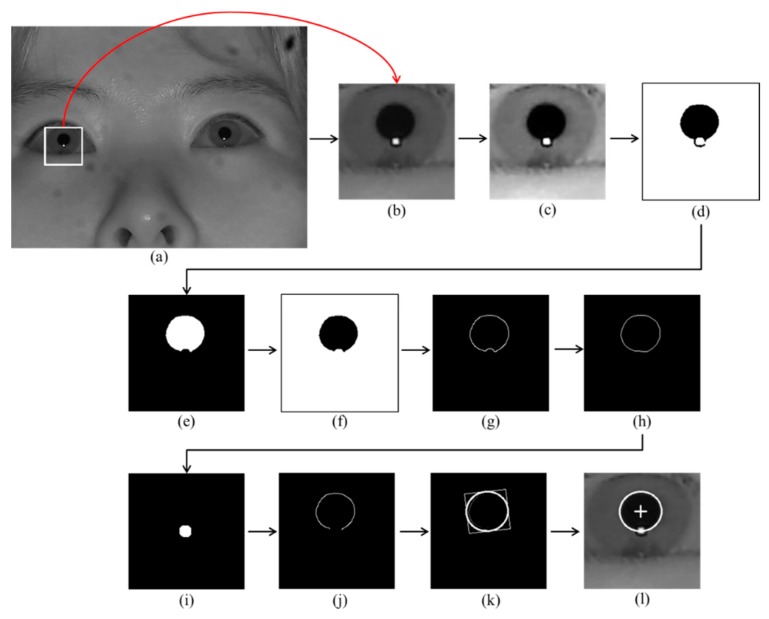
Pupil center region detection process: (**a**) eye detection result based on corneal specular reflection from the original image, (**b**) eye region detected from the original image; (**c**) application of histogram stretching to the image of (b); (**d**) application of binarization to the image of (c); (**e**) application of morphology calculation to the image of (d); (**f**) application of component labeling to the image of (e); (**g**) application of canny edge detection to the image of (f); (**h**) application of a convex hull to the image of (g); (**i**) image that has binarized the reflection with the image of (c); (**j**) image that has removed the overlapped parts of (h,i) from the image of (h); (**k**) application of ellipse-fitting algorithm to the image of (j); and (**l**) the final detection result of the pupil center region.

The rough corneal specular reflection of the user is detected based on the bright pixel values in the image. Based on this reflection, the eye region of the user is detected, as shown in Figure 1 step (1) and Figure 2b. The image in Figure 2c is a result of applying a histogram stretching calculation to the image of Figure 2b to obtain a binarization result for a clearer distinction. The image in Figure 2d is a result of applying a binarization calculation to the image in Figure 2c. The image in Figure 2e is a result of removing small noises by applying a morphology calculation to the image in Figure 2d. The image in Figure 2f is a result of leaving only the image of the pupil shape by applying a component labeling to the image in Figure 2e. As shown in the image of Figure 2g, the edge of the pupil shape was detected by applying canny edge detection to the image in Figure 2f. In addition, applying a convex hull method [20,21,22] to the image in Figure 2g, the image in Figure 2h shows the result of compensating the pupil part damaged from the reflection occurring on the pupil. The image in Figure 2i is a binarized version of the image in Figure 2c centering on the reflection. Figure 2j shows an image, in which the overlapped part of Figure 2i,h images is removed from the image of Figure 2h. In the image in Figure 2j, because the pupil boundary and the corneal specular reflection boundary are touching each other, a concave portion is detected during the pupil boundary detection. Then, a shape different from the real pupil boundary is detected. To resolve this, we remove the overlapped parts in Figure 2h,i from the image in Figure 2h. Then, the pupil boundary that has removed the touching part can be detected, as shown in the image in Figure 2j. Figure 2k shows the detection of the final pupil boundary by applying an ellipse-fitting algorithm using the image in Figure 2j. Figure 2l shows the final resulting image.

Based on the detected position of the pupil center, the searching area for detecting corneal specular reflection is defined. Then, the center of corneal specular reflection is located based on binarization and calculating the geometric center within this area.

#### 2.1.2. Calculating the User’s Gaze Position

**Figure 3:**
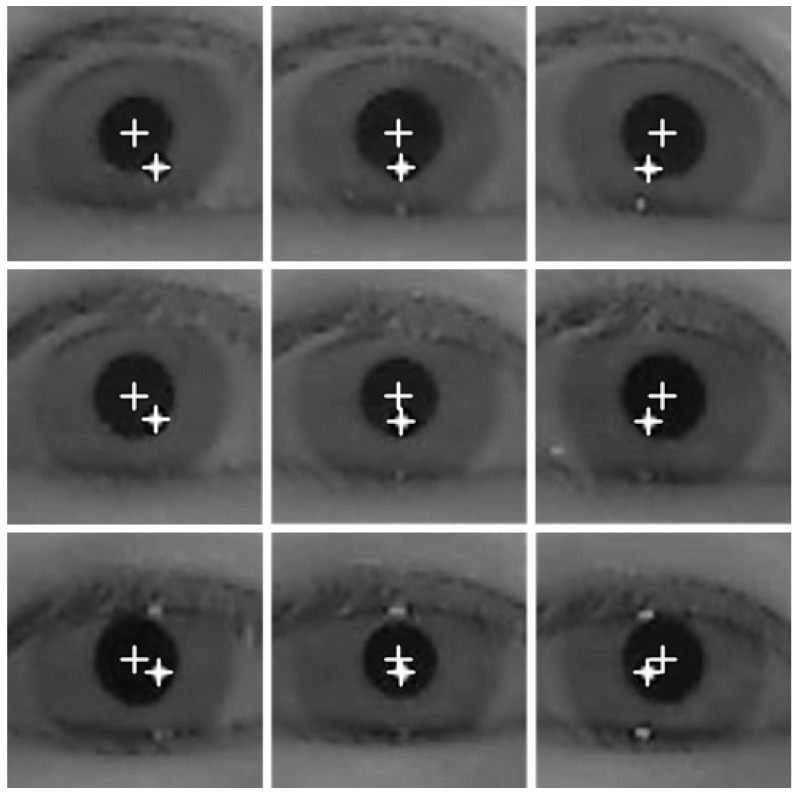
Images of user’s eye region when gazing at nine calibration points on the screen.

**Figure 4:**
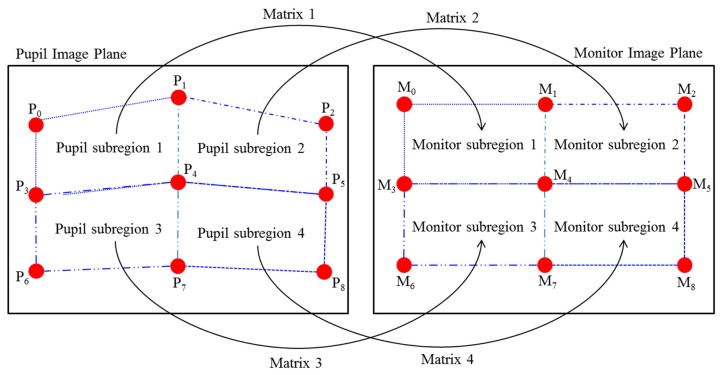
Corresponding relations of four subregions defined by nine pupil centers and four subregions in a monitor defined using calibration points.

The gaze position of the user is calculated using the detected pupil center and reflection center coordinates. To compensate the differences in each person’s eyeball and cornea size, as well as the angle kappa, and to find the relationship between the user’s eye and screen coordinates, the calibration process is conducted before the gaze position is calculated. The gaze position is calculated using several geometric transform matrices obtained through the calibration process [19]. When nine reference points are gazed at during the calibration, by using each of the nine pupil center positions obtained as shown in Figure 3, the relationship between the screen and pupil center can be expressed as in Figure 4. As Figure 4 illustrates, when the user is looking at the nine calibration points, the pupil image plane can be mainly divided into four subregions by using the center position of the pupil. Each of the four pupil subregions can be mapped to four monitor subregions, which are divided into the nine calibration points of the monitor image plane. In addition, a geometric transform matrix corresponding to each subregion is obtained and used [19,23]. For example, pupil subregion 3 can be mapped into monitor subregion 3, and here, matrix 3 is used as shown in Figure 4.

**Figure 5:**
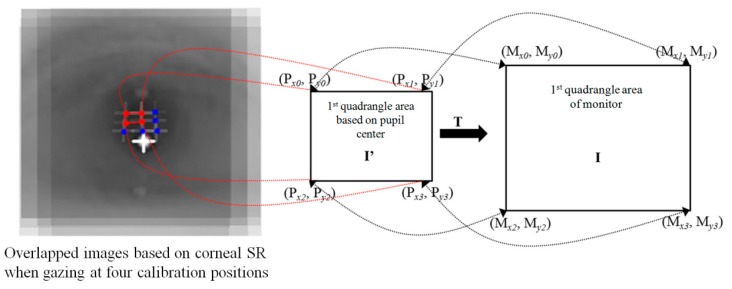
Example of correspondence between four pupil centers and four monitor corners when a user is gazing at the four corners of a monitor.

When calculating the user’s gaze position, if the user’s pupil center exists at pupil subregion 2 (P_2_P_3_P_6_P_5_), the user’s gaze position is calculated using matrix 2 among the four geometric transforms. Here, the gaze position is calculated through the processes in Equations (1) and (2) [19]. Figure 5 shows the example of correspondence between four pupil centers and four monitor corners when a user is gazing at the four corners of a monitor.

(1) $$\left[\begin{array}{c} \begin{array}{cccc} {\text{M}}_{\text{x}0} & {\text{M}}_{\text{x}1} & {\text{M}}_{\text{x}2} & {\text{M}}_{\text{x}3} \end{array}\\ \begin{array}{cccc} {\text{M}}_{\text{y}0} & {\text{M}}_{\text{y}1} & {\text{M}}_{\text{y}2} & {\text{M}}_{\text{y}3} \end{array} \end{array}\right]=\left[\begin{array}{c} \begin{array}{cccc} \text{a} & \text{b} & \text{c} & \text{d} \end{array}\\ \begin{array}{cccc} \text{e} & \text{f} & \text{g} & \text{h} \end{array} \end{array}\right]\left[\begin{array}{cccc} {\text{P}}_{\text{x}0} & {\text{P}}_{\text{x}1} & {\text{P}}_{\text{x}2} & {\text{P}}_{\text{x}3}\\ {\text{P}}_{\text{y}0} & {\text{P}}_{\text{y}1} & {\text{P}}_{\text{y}2} & {\text{P}}_{\text{y}3}\\ {\text{P}}_{\text{x}0}{\text{P}}_{\text{yo}} & {\text{P}}_{\text{x}1}{\text{P}}_{\text{y}1} & {\text{P}}_{\text{x}2}{\text{P}}_{\text{y}2} & {\text{P}}_{\text{x}3}{\text{P}}_{\text{y}3}\\ 1 & 1 & 1 & 1 \end{array}\right]$$

(2) $$\left[\begin{array}{c} {{\text{M}}^{\prime }}_{\text{x}}\\ {{\text{M}}^{\prime }}_{\text{y}} \end{array}\right]=\left[\begin{array}{c} \begin{array}{cccc} \text{a} & \text{b} & \text{c} & \text{d}\\ \text{e} & \text{f} & \text{g} & \text{h} \end{array} \end{array}\right]\left[\begin{array}{c} {{\text{P}}^{\prime }}_{\text{x}}\\ {{\text{P}}^{\prime }}_{\text{y}}\\ {{\text{P}}^{\prime }}_{\text{x}}{{\text{P}}^{\prime }}_{\text{y}}\\ 1 \end{array}\right]$$

### 2.2. User’s Gaze Tracking Estimation Using a Fuzzy System

#### 2.2.1. Three Features for the Inputs of Fuzzy System, and Fuzzy Membership Function with Rule Table

**Figure 6:**
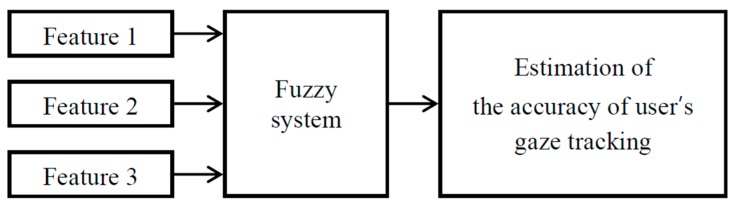
Process of estimating the gaze tracking accuracy of the user by applying a fuzzy algorithm.

In this study, a method for estimating a user’s gaze tracking accuracy is proposed using a fuzzy system based on information obtained during the calibration process. As shown in Figure 6, the gaze tracking accuracy of the user is estimated based on the fuzzy algorithm by using three feature values of the user, which can be obtained during the calibration process.

**Figure 7:**
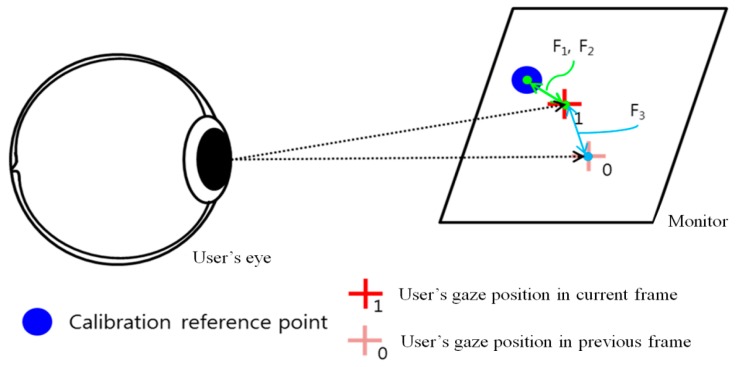
The feature values that can be obtained during the calibration process.

Among the eye features of the user that can be obtained during the calibration process, the following three feature values were utilized. In Figure 7, the Euclidean distances are calculated between the reference points that have to be looked at during the calibration, and the positions that the user is actually gazing at. Then, Feature 1 (F_1_) indicates the mean value of the Euclidean distances. Feature 2 (F_2_) indicates the standard deviation of the Euclidean distances. Feature 3 (F_3_) indicates the amount of gaze position movement of the user between the previous and current image frames.

**Figure 8:**
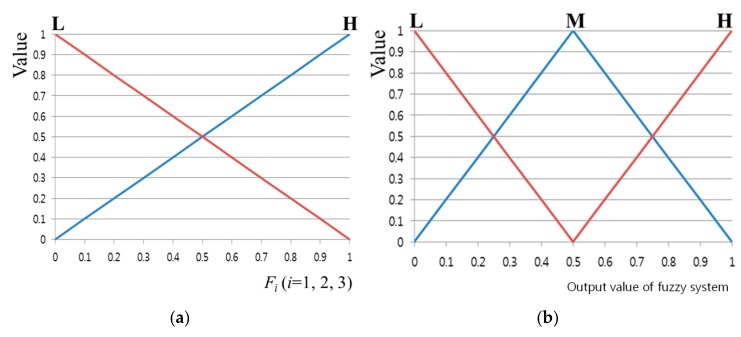
Membership functions of calibration-information based gaze accuracy estimation system: (**a**) input and (**b**) output membership functions.

With respect to the use of the fuzzy algorithm, the design of the membership function is a very important factor. Figure 8 shows the input/output membership function configuration of the feature values. As shown in Figure 8, a linear member function or triangular membership function is most commonly used because the system’s calculation speed is fast and the complexity of the codes can be relatively reduced [24,25,26]. Features 1–3 described above each show different values, and the range of each feature value is different. Furthermore, depending on the user’s calibration result, the ranges of Features 1–3 change irregularly. Therefore, each feature value was normalized with a value of zero to 1, as shown in Figure 8. The fuzzy membership functions can be mainly divided into two types. As shown in Figure 8a, an input membership function having low (L) and high (H) values was designed. In addition, as shown in Figure 8b, an output membership function having low (L), middle (M), and high (H) values was designed.

Furthermore, a fuzzy rule table (Table 2) was designed. For example, if the mean and standard deviation values are small for the Euclidean distance values between the reference and user’s gaze positions, and if the amount of gaze movement of the user is small between image frames, it can be regarded that the error of the user’s gaze position is also small. In other words, if the values of Features 1–3 are L, then the error value of the gaze tracking can be estimated to be L as well. If the values of Features 1–3 are high (H), then the error value of the gaze tracking can be also estimated to be high (H).

**Table 2:** Fuzzy rule table.

Feature 1	Feature 2	Feature 3	Output
L	L	L	L
		H	L
	H	L	L
		H	M
H	L	L	M
		H	H
	H	L	H
		H	H

#### 2.2.2. Obtaining the Output of Fuzzy System Using Defuzzification Methods

In general, an output value of the fuzzy system can be obtained through the output fuzzy membership function and the fuzzy rule. The input function in Figure 8a has two output values and one input value (*F_i_*). In other words, because there are three input feature values, there are a total of eight output values.

**Figure 9:**
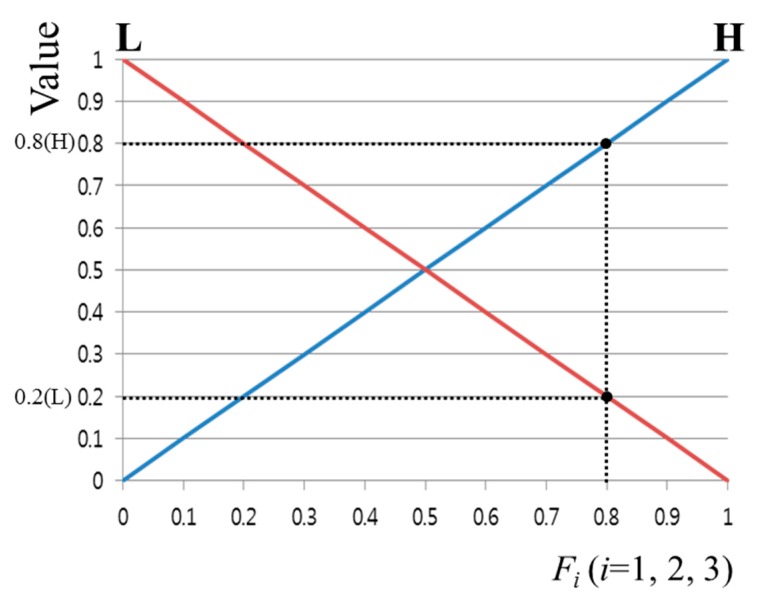
Example of obtaining an output value when one input value is input into the input membership function.

For example, as can be seen in Figure 9, the input value of 0.8 has output values of 0.2(L) and 0.8(H). These two output values are because of Feature 1, but also apply to Features 2 and 3. This means that there are three pairs of output values, {0.2 (L), 0.8 (H)}. Based on these three pairs of output values, eight combinations can be obtained: {0.2(L), 0.2(L), 0.2(L)}, {0.2(L), 0.2(L), 0.8(H)}, {0.2(L), 0.8(H), 0.2(L)} … {0.8(H), 0.8(H), 0.8(H)}. Whenever a single pair of combination values is elicited, one of the values can be determined from (0.2, 0.8) and (L, M, H) through the Min rule, Max rule, and fuzzy rule table [27]. For example, when there is a combination of {0.2(L), 0.8(H), 0.8(H)}, a value of 0.2 can be determined by the Min rule. In addition, in Table 2, because the output value is M when Feature 1 is L and Features 2 and 3 are H, the value of M can also be determined by the fuzzy rule. That is, from the values of {0.2(L), 0.8(H), 0.8(H)}, an inference value of 0.2(M) can be obtained by the Min and fuzzy rules. If the Max rule is used, an inference value of 0.8(M) can be obtained. As mentioned above, because there are a total of eight combinations, the number of inference values is also eight in total.

**Figure 10:**
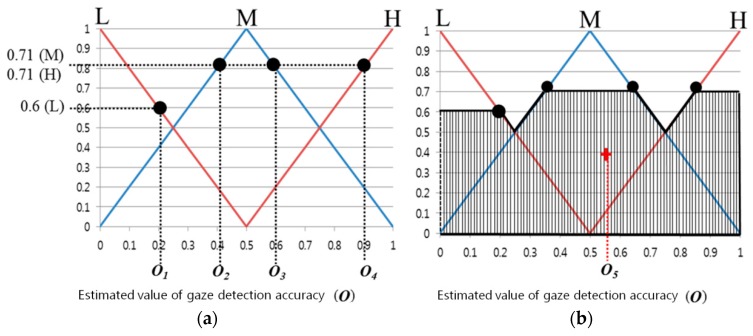
Examples of output value results through various defuzzification methods when an inference value is input: (**a**) FOM, LOM, MOM, MeOM, and (**b**) COG.

In Figure 10a, several output values can be obtained using one inference value. For example, when the inference value is 0.71(M), the output values *O_2_*, *O_3_*, and *O_4_* can be obtained. From these several output values, using a defuzzification method, one final output value can be determined [28,29]. This output value is used as the accuracy estimation score of the gaze tracking system.

Conventionally, various defuzzification methods exist, and the performances of five methods were compared: First of Maxima (FOM), Last of Maxima (LOM), Middle of Maxima (MOM), Mean of Maxima (MeOM), and Center of Gravity (COG) [28,29]. FOM selects the first value in the output values calculated with the largest value among the inference values obtained. LOM selects the last value in the output values calculated with the largest value among the inference values obtained. MOM selects the median of the first and last values from the output values calculated with the largest value among the inference values obtained [28]. MeOM selects the mean of the output values calculated with the largest value among the inference values obtained. The final output values obtained according to each method in Figure 10a,b are *O_2_* for FOM, *O_4_* for LOM, ((*O_2_* + *O_4_*)/2) for MOM, ((*O_2_* + *O_3_* + *O_4_*)/3) for MeOM, and *O_5_* for COG. COG is determined through the mass center point of the region expressed with the slashes shown in Figure 10b.

## 3. Experiment Results and Discussion

**Figure 11:**
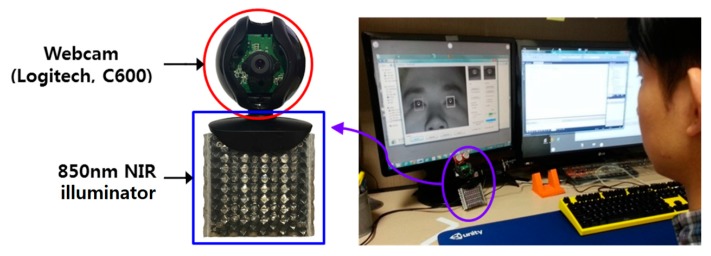
The gaze tracking system and experimental environment used in this study.

Figure 11 shows the experimental environment used in this study. For the experiment, a desktop computer containing a 3.5-GHz Intel^®^ core™ i7-3770K CPU and 8 GB of RAM was used. The monitor resolution was 1280 × 1024. For the camera, a 700-nm long pass filter (Kodak Wratten Filter No. 89B, Rochester, NY, USA [30]) and a zoom lens were mounted on a webcam (Logitech C600, Lausanne, Switzerland [31]) having a universal serial bus (USB) interface. In addition, an 850-nm reference illuminator was used in an 8 × 8 array. The camera was positioned right below the center of the monitor, and the illuminator was also positioned below the camera. The distance between the monitor and user was roughly 75 cm. Our program was implemented using a Microsoft foundation class based C++ program, DirectX 9.0 SDK, and OpenCV in a Microsoft Visual Studio C++ 2010 development environment.

The calibration was carried out according to each marker type and display method described in Section 3.1. The gaze position accuracy of the user was measured for each calibration. The experiment was repeated four times for each test subject and a total of seven calibration methods. A total of 15 test subjects were used.

### 3.1. Various Calibration Marker Types and Display Methods

In this paper, various calibration marker types and display methods are proposed to check the validity of the user-calibration based gaze tracking estimation model. As shown in Figure 1, the calibration marker types are classified into two types: static marker and dynamic marker types.

**Figure 12:**
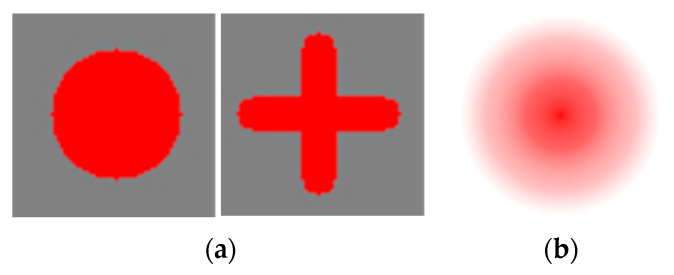
Various calibration marker types: (**a**) static and (**b**) dynamic markers.

**Figure 13:**
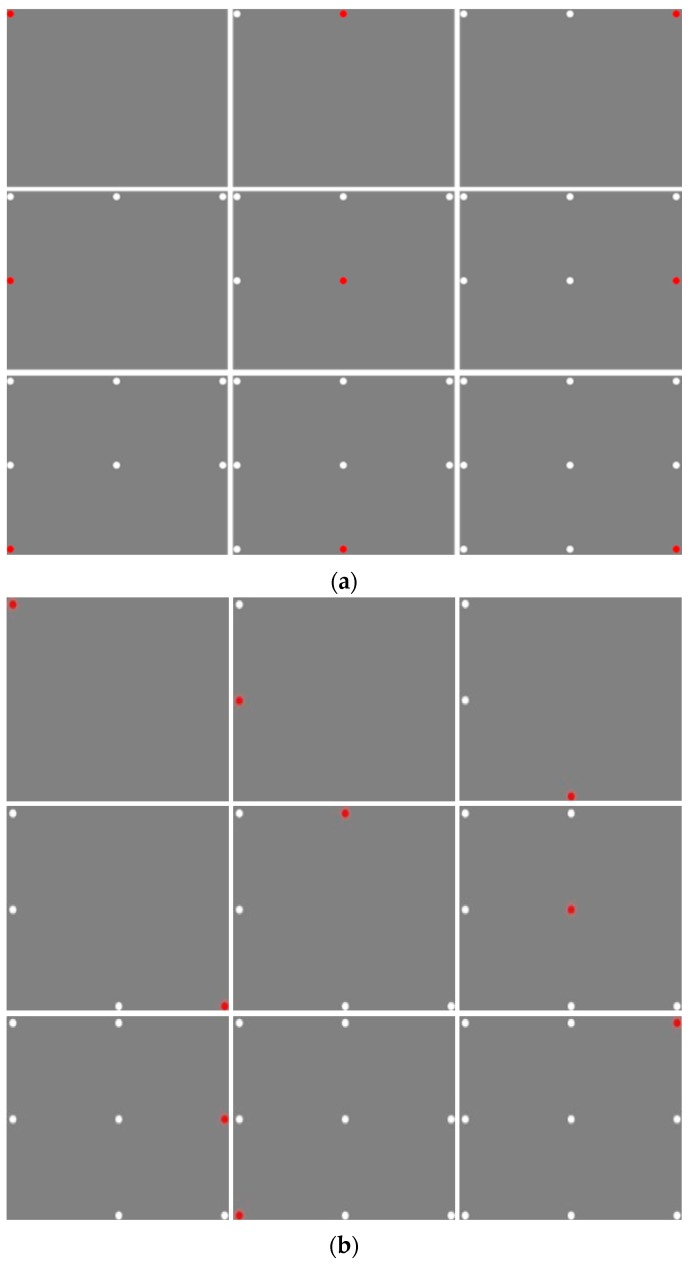
Marker display methods: (**a**) sequential and (**b**) random marker displays.

**Figure 14:**
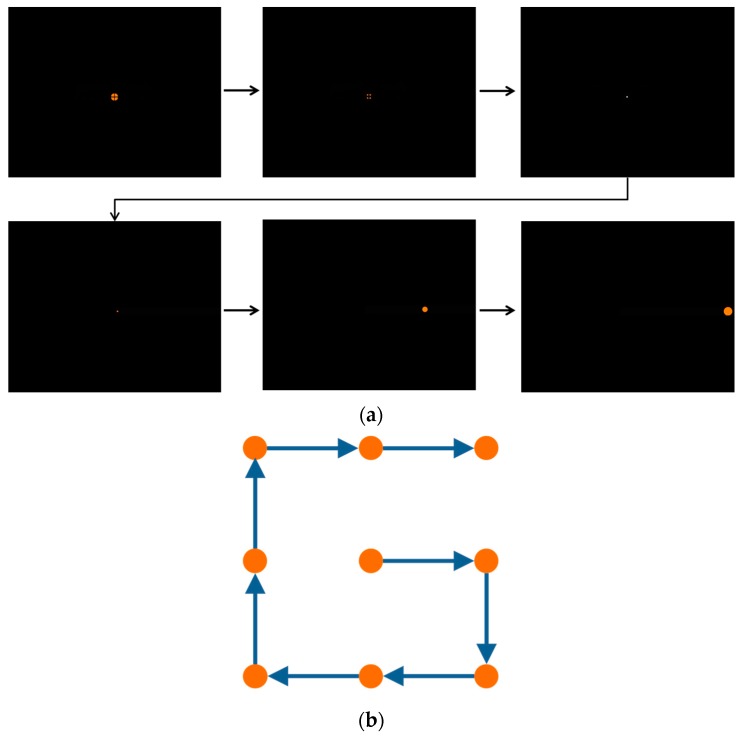
Guiding display method: (**a**) an expression guiding to the next marker position of the calibration, and (**b**) an example of a marker’s movement path.

Among the commonly used calibration markers, Figure 12a shows the static markers of a circle shape and a cross. Figure 12b shows a dynamic marker with a dynamically changing shape, *i.e.*, the color is becoming darker gradually as the shape of the marker decreases from a large circle to a small circle in the actual system. In addition, using each marker, the calibration was performed with different display methods. The marker display methods include a commonly used sequential display method shown in Figure 13a, and a random display method shown in Figure 13b. In addition, they include the method of dynamically guiding to the next display position of the calibration marker, named as guiding display method, shown in Figure 14.

In Figure 13, the red dot is a calibration point that the user has to gaze at, and, in the next step, the calibration finishes with point represented by a white color.

**Figure 15:**
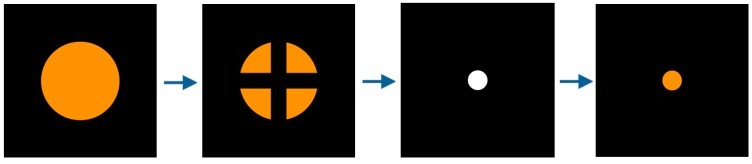
Changes to dynamic marker in the guiding display.

In Figure 14, a dynamic marker is used, and the marker shape changes step by step, as shown in Figure 15. At first, the marker is expressed as shown in the leftmost image of Figure 15, and afterward, the marker changes as shown in the images on the right-hand side. As shown in the second image from the left in Figure 15, the marker is divided into four points and disappears. And, as shown in the third image from the left in Figure 15, at the moment of calibration it blinks as a white dot. Afterward, as shown in the rightmost image of Figure 15, it is changed to an orange dot, and while moving to the next calibration point, it is increased to the marker size shown in the leftmost image of Figure 15. As the marker shape changes in this manner, the calibration is performed at a total of nine positions, as shown in the sequence in Figure 14.

### 3.2. Calibration Results and Gaze Detection Accuracy

**Figure 16:**
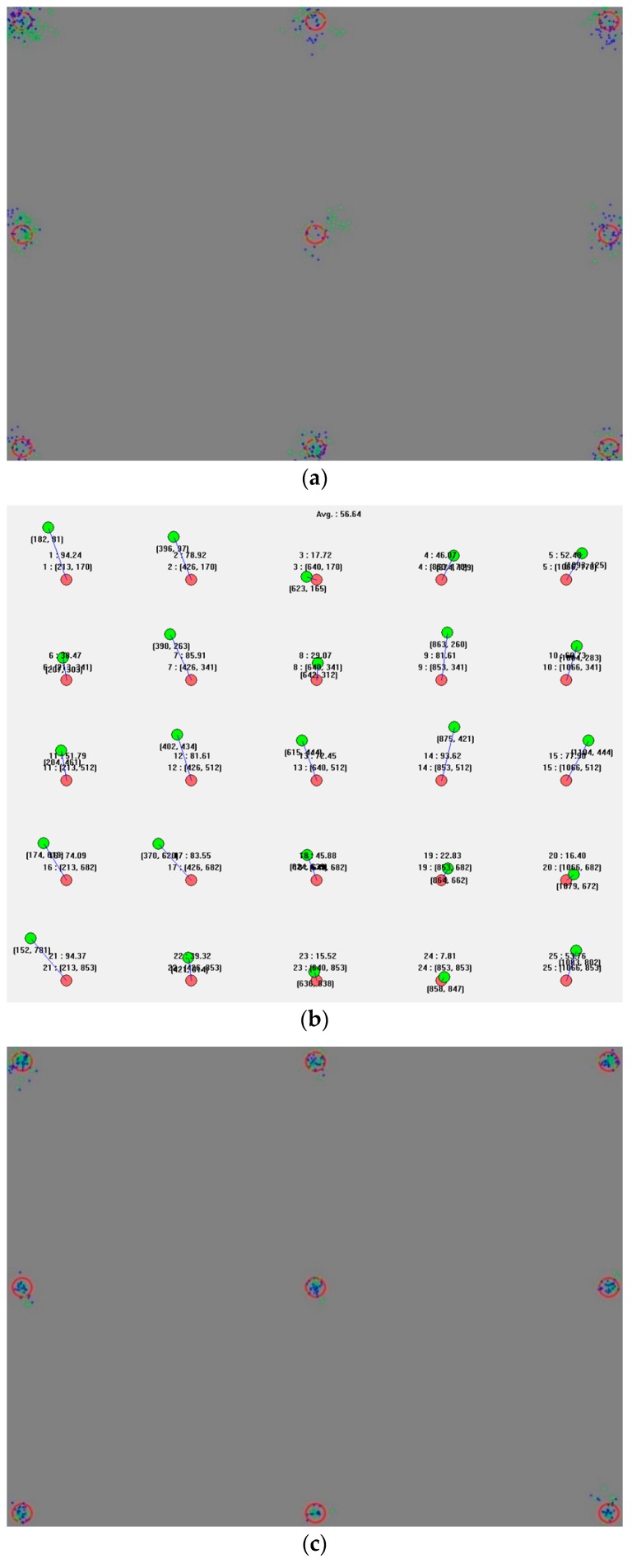

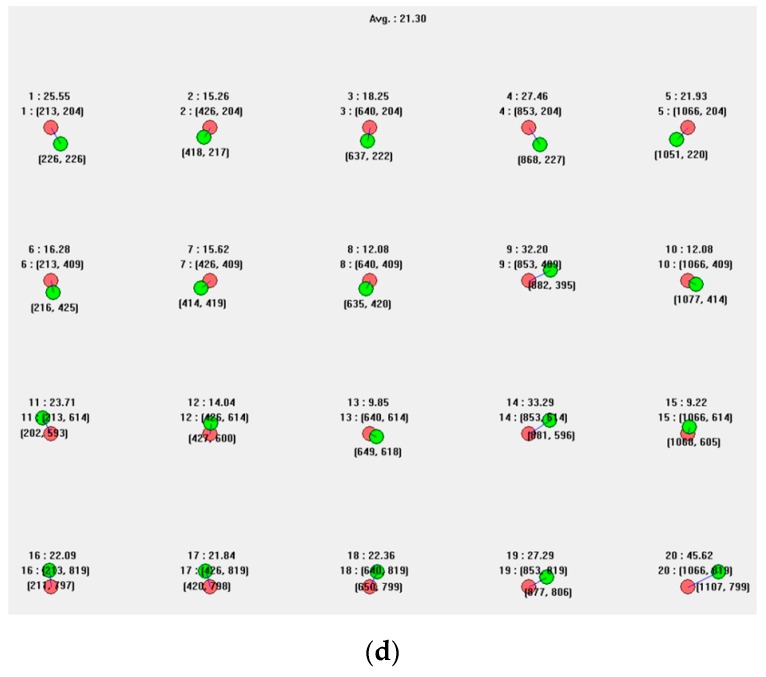
Results of a circular marker type expressed sequentially: (**a**) an inaccurate calibration, (**b**) an error in the gaze tracking accuracy of 1.223°; (**c**) an accurate calibration; and (**d**) an error in the gaze tracking accuracy of 0.407°.

**Figure 17:**
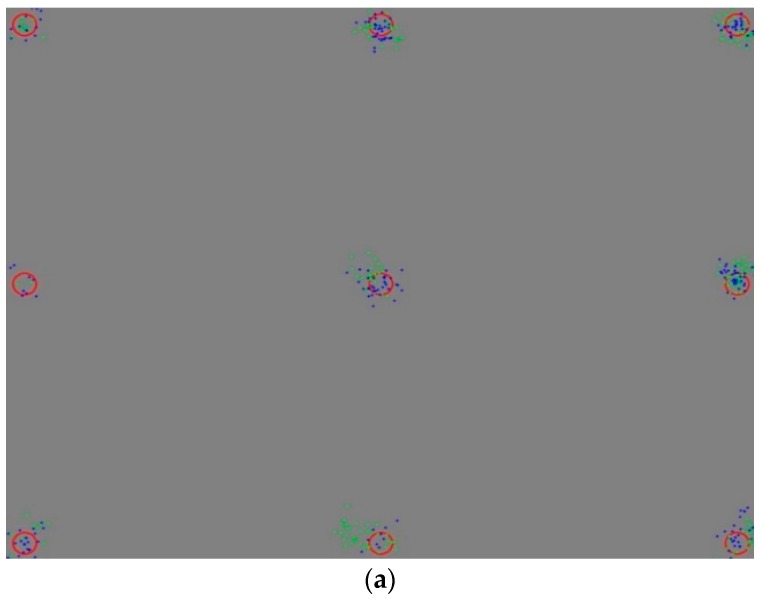

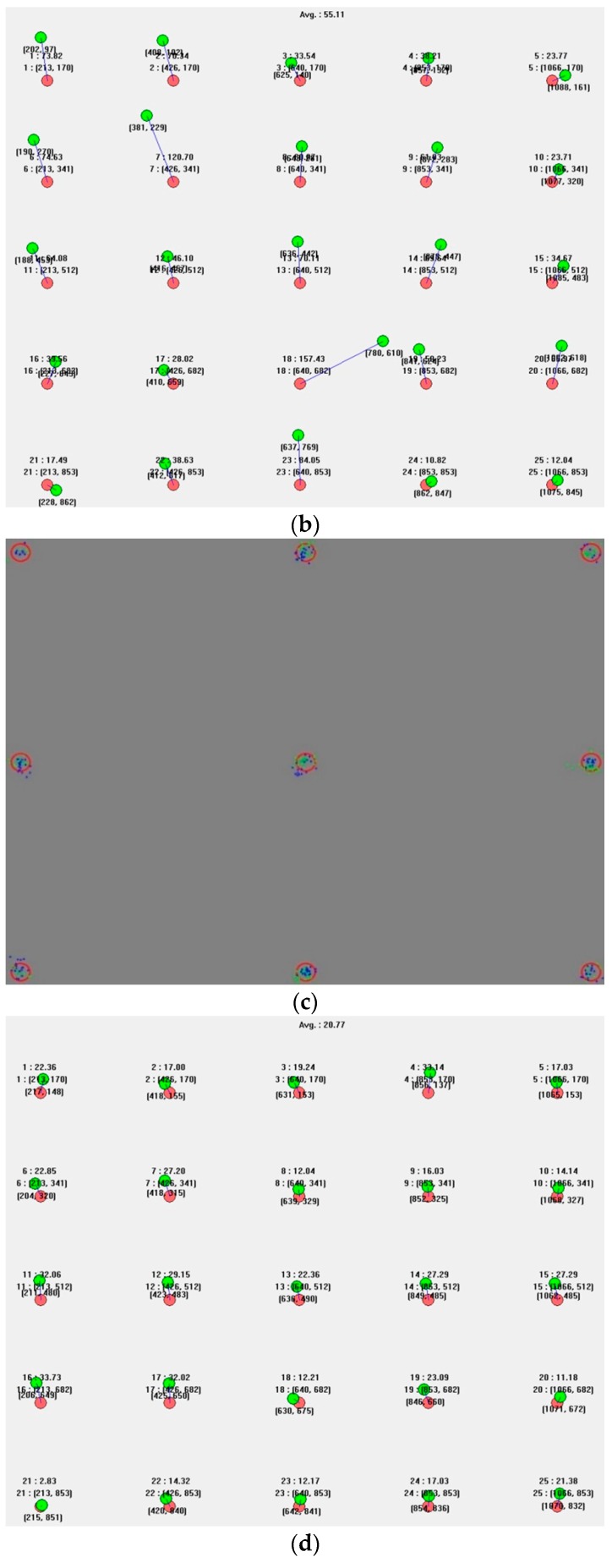
Results of a cross-shaped marker type expressed sequentially: (**a**) an inaccurate calibration, (**b**) an error in the gaze tracking accuracy of 1.19°; (**c**) an accurate calibration; and (**d**) an error in the gaze tracking accuracy of 0.449°.

**Figure 18:**
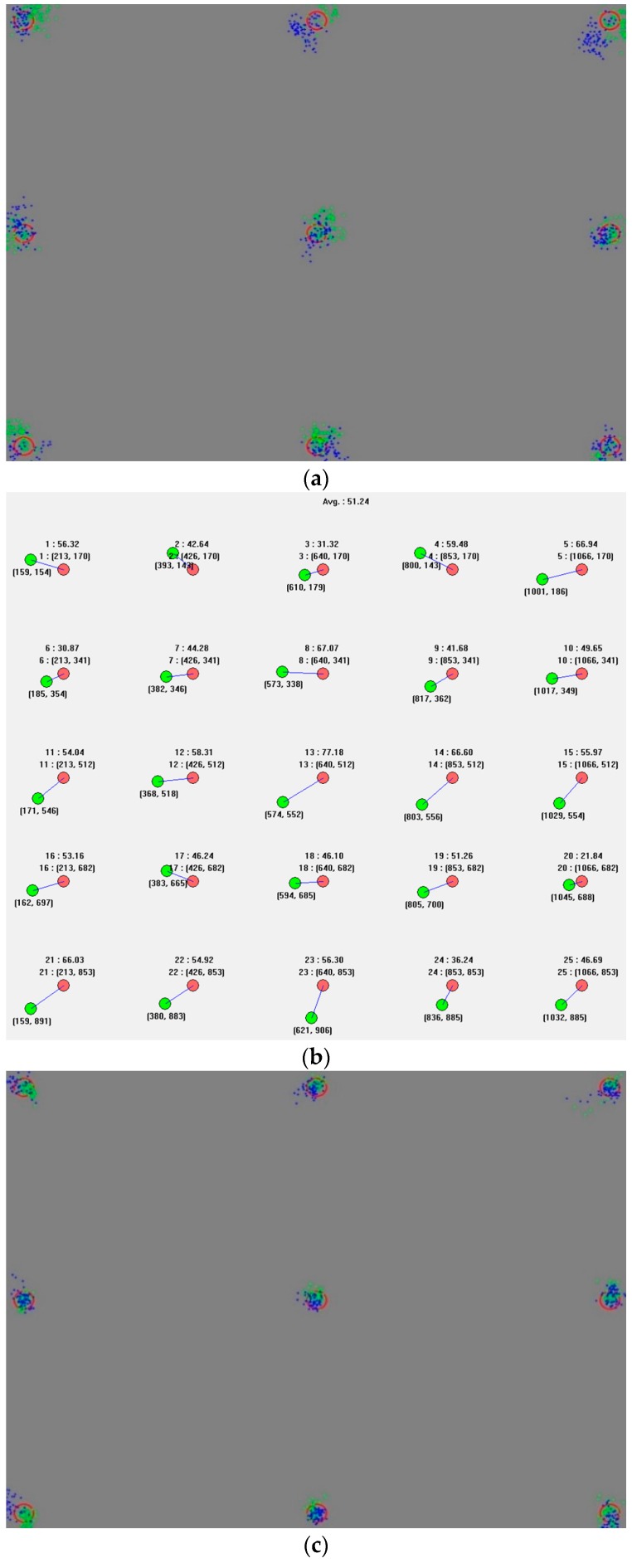

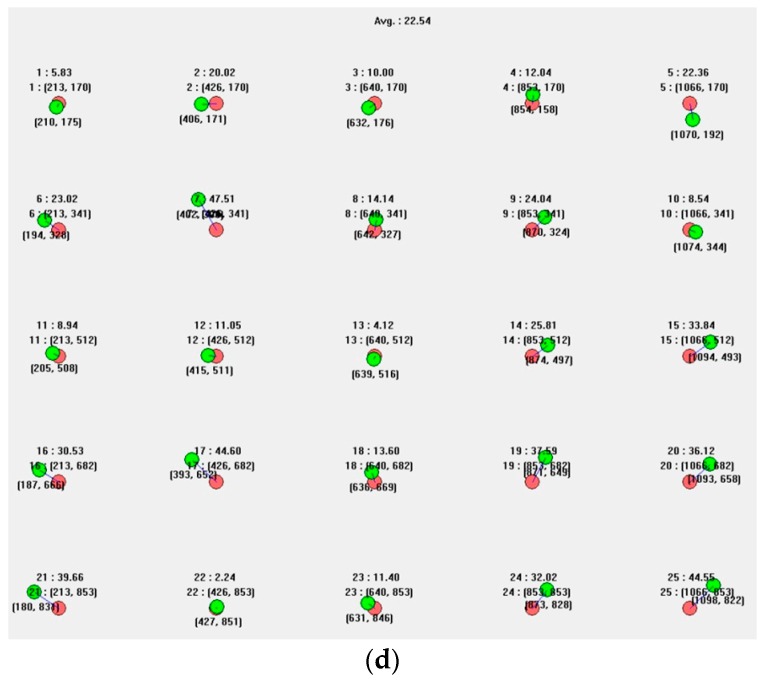
Results of a gradation marker type expressed sequentially: (**a**) an inaccurate calibration, (**b**) an error in the gaze tracking accuracy of 1.107°; (**c**) an accurate calibration; and (**d**) an error in the gaze tracking accuracy of 0.487°.

In this section, we show the user calibration results and gaze tracking accuracy. In Figure 16, Figure 17 and Figure 18, the blue and green dots of (a,c) mark the gaze coordinates of both eyes during the user calibration. The red circles represent the regions that have to be looked at. As shown in (a,c) of Figure 16, Figure 17 and Figure 18, when the calibration gaze coordinates are concentrated in regions that have to be looked at during the calibration, the error in the gaze tracking accuracy of the users is small, as shown in (b,d). Based on this, it can be seen that the accuracy levels during the user calibration and gaze tracking are related. At the same time, it is confirmed that the gaze tracking estimation model can be obtained using the proposed three features. The first and second features are respectively the mean and standard deviations of the Euclidean distances between the user’s calculated gaze and the reference positions obtained during the user calibration. The third one is the amount of gaze movement between the previous and current image frames.

### 3.3. Estimation Analysis of Fuzzy Algorithm for User Gaze Tracking Accuracy Using Calibration Information

In this study, a method for estimating the user’s gaze tracking accuracy was proposed by applying a fuzzy algorithm to three feature values obtained during the user calibration. From Table 3, the gaze tracing accuracy estimation results can be confirmed according to the fuzzy system’s Min and Max rules and various defuzzification methods. In the results shown in Table 3, it was found that the estimation value of the fuzzy system obtained when the Min rule and COG were used had the highest correlation with the size of the real gaze error. In other words, when the estimation of the fuzzy system was small, the real gaze error was small. In addition, when the estimation of the fuzzy system was large, the real gaze error was large.

**Table 3:** Estimation results of gaze tracking accuracy according to the marker type and display method (Seq.: Sequential display, Rand.: Random display).

Display Method			Seq.	Rand.	Moving	Seq.	Rand.	Seq.	Rand.
Marker Type			Cross	Cross		Circle	Gradation	Gradation	Circle
Real Gaze Error (°)			0.6363	0.6742	0.684	0.7527	0.7697	0.8371	0.8498
Fuzzy system	Min rule	FOM	0.255	0.2299	0.3918	0.3708	0.4461	0.4408	0.1399
		MOM	0.255	0.2768	0.4086	0.4332	0.2763	0.3271	0.1488
		LOM	0.255	0.3237	0.4254	0.4957	0.3422	0.3143	0.1576
		MeOM	0.1948	0.1991	0.2622	0.2671	0.5737	0.5796	0.1259
		COG	**0.3764**	**0.4177**	**0.4688**	**0.4859**	**0.3018**	**0.7411**	**0.3198**
	Max rule	FOM	0.1107	0.1231	0.1451	0.1106	0.4438	0.2446	0.0931
		MOM	0.5357	0.5278	0.5032	0.5224	0.4165	0.4518	0.4136
		LOM	0.9607	0.9324	0.8613	0.9341	0.2692	0.2854	0.7341
		MeOM	0.3122	0.3032	0.3124	0.3201	0.464	0.6109	0.2228
		COG	0.5002	0.5002	0.4991	0.5002	0.5006	0.3433	0.4988

**Figure 19:**
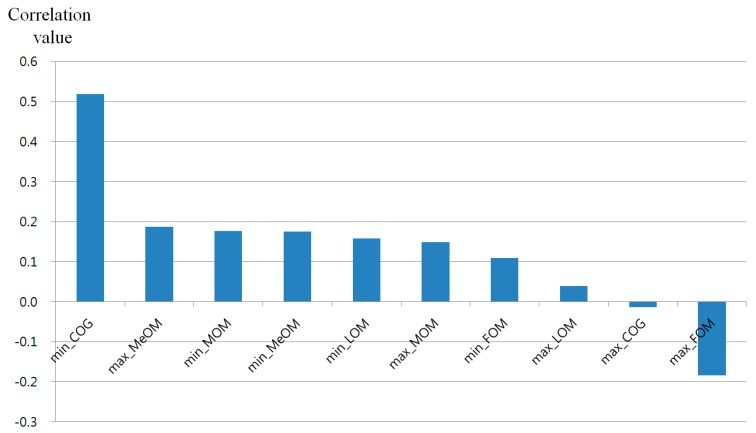
The correlation values between the real gaze errors and each method of defuzzification using the Min or Max rule.

To find the correlation between the real gaze error and each method of defuzzification with the Min or Max rule, the correlation, gradient, and R^2^ values were calculated, as shown in Table 4. The range in correlation values was −1 to 1, indicating a negative or positive correlation among the real data. A correlation value of zero indicates that there is no correlation between two sets of data. As shown in Table 4 and Figure 19, when the Min rule and COG were used, the correlation value was the highest between the real gaze error data and the fuzzy system’s output estimation value.

Most of the two-dimensional data can be expressed through a linear fitted line, which can express the distribution, and can be calculated using the gradient and R^2^. Here, R^2^ shows reliability between the data distribution and a linear line. Its value increases as the data are distributed closer to the linear fitted line [32]. As shown in Table 4, when the COG of the Min rule was used, the gradient was closest to 1 and the value of R^2^ was the largest. Based on these results, it was found that using the COG method of the Min rule is best when a user is estimating a gaze error.

**Table 4:** The correlations between the real gaze tracking errors and the estimation values for each method of defuzzification using the Min or Max rule.

Method			Correlation Value	Gradient	R^2^
Fuzzy system	Min rule	FOM	0.1093	0.5177	0.068
		MOM	0.1765	0.8036	0.1307
		LOM	0.158	0.7269	0.1464
		MeOM	0.1756	0.5775	0.0776
		COG	0.5184	1.5771	0.2593
	Max rule	FOM	−0.1841	−0.3851	0.1827
		MOM	0.1481	0.3375	0.0784
		LOM	0.0393	0.2843	0.0078
		MeOM	0.1872	0.8416	0.1558
		COG	−0.0134	0.0848	0.0963

The following is a statistical analysis of the differences between correlation values according to the correlation value ranking and each method of defuzzification using the Min or Max rule.

**Figure 20:**
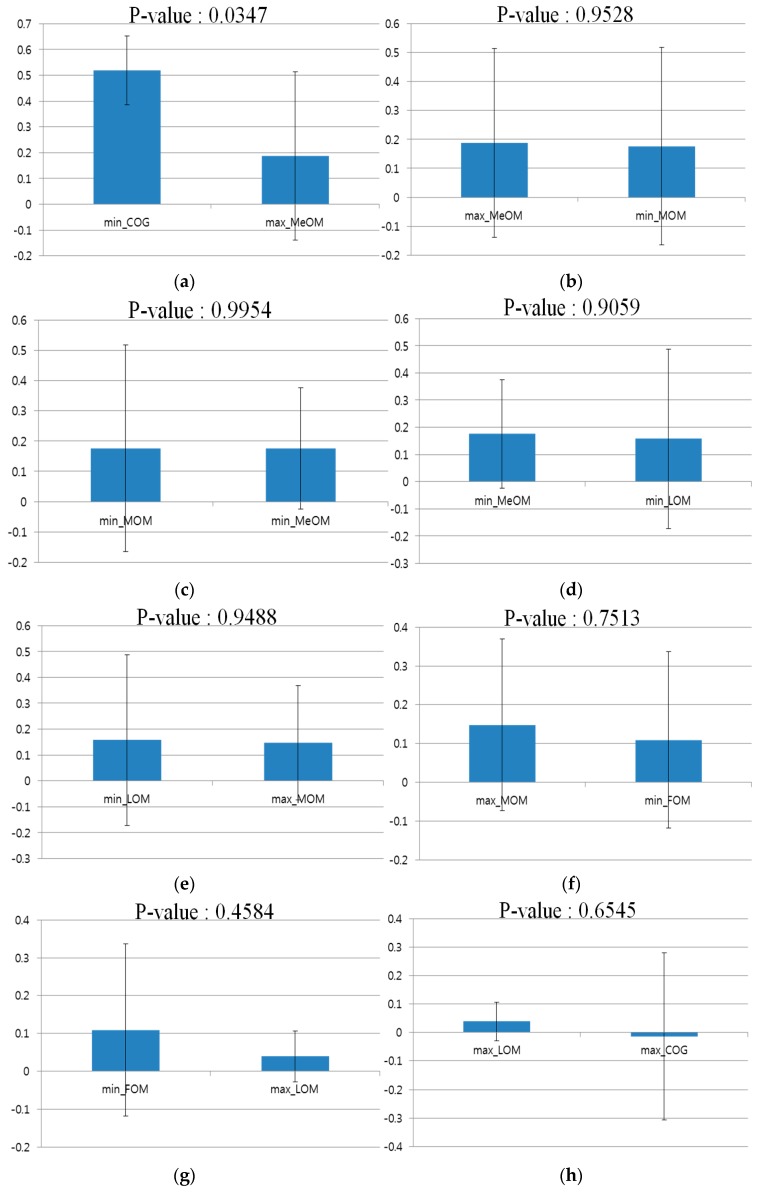

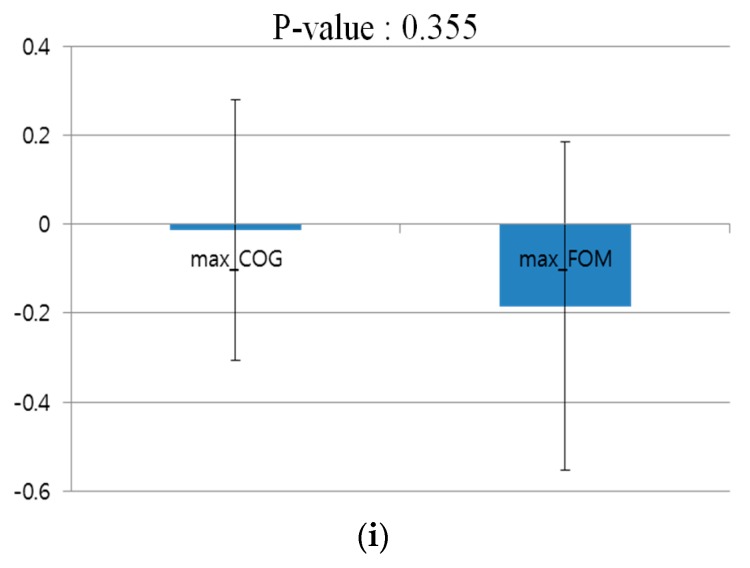
A *t*-test between defuzzification methods based on the correlation value ranking of Figure 19: *t*-test between ranks (**a**) 1 and 2, (**b**) 2 and 3; (**c**) 3 and 4; (**d**) 4 and 5; (**e**) 5 and 6; (**f**) 6 and 7; (**g**) 7 and 8; (**h**) 8 and 9; and (**i**) 9 and 10.

As shown in Table 4 and Figure 19, the difference between the COG of the Min rule and MeOM of the Max rule, which ranked 1 and 2 on the basis of the correlation values, respectively, was checked through a *t*-test [33]. This is a statistical hypothesis test commonly used in statistical analysis. When the *t*-test was performed using two independent samples, the COG of the Min rule and MeOM of the max rule, the calculated *p*-value was 0.0347, which was smaller than the 95% (0.05) significance level. That is, because the null-hypothesis that there is no difference between two correlation values is rejected, it indicates that there is a significant difference in the correlation values between the two groups, *i.e.*, the COG of the Min rule and the MeOM of the Max rule. This shows that, to estimate the gaze error of the user, it is appropriate to use the COG of the Min rule, which has the top ranking correlation value among the defuzzification methods. As shown in Figure 20b–i, in all other cases of the ranked correlation values, *i.e.*, the No. 2 and 3 through No. 9 and 10 rankings, the *p*-value was larger than 0.05, which does not show a statistically significant difference.

If the null-hypothesis between the samples is determined using the *p*-value through a *t*-test, the size of the difference between the two groups can be shown using the size of the effect. This can be defined as Cohen’s d [34]. Cohen’s d is considered as small at around 0.2–0.3, medium at around 0.5, and large at 0.8 or higher. For example, in Table 5, in the case of the COG of the Min rule and MeOM of the Max rule, Cohen’s d is 1.2425. Through this, it can be stated that the difference in the correlation values is very large between the COG of the Min rule and MeOM of the Max rule, to the point of having a large effect size. It can be seen from the *p*-value and Cohen’s d that the difference is large between the COG of the Min rule and MeOM of the Max rule, which have No. 1 and 2 ranked correlation values, respectively. Through this, when estimating the gaze position error of the user, it was found to be most appropriate to use the COG method of the Min rule.

**Table 5:** Cohen’s d and effect size calculated between each fuzzy method sequentially according to the correlation value rankings.

Defuzzification Methods with Min or Max Rule	*p*-Value	Cohen’s d	Effect Size
min_COG *vs.* max_MeOM	0.0347	1.2425	Large
max_MeOM *vs.* min_MOM	0.9528	0.0301	Small
min_MOM *vs.* min_MeOM	0.9954	0.003	Small
min_MeOM *vs.* min_LOM	0.9059	0.0604	Small
min_LOM *vs.* max_MOM	0.9488	0.0328	Small
max_MOM *vs.* min_FOM	0.7513	0.1616	Small
min_FOM *vs.* max_LOM	0.4584	0.3895	Small
max_LOM *vs.* max_COG	0.6545	0.2324	Small
max_COG *vs.* max_FOM	0.355	0.4796	Medium

## 4. Conclusions

In this study, a fuzzy-based estimation model was proposed to measure the accuracy of a gaze tracking system based on the user’s calibration information. The accuracy of the gaze tracking system was estimated based on three types of feature values. The first and second features are respectively the mean and standard deviations of the Euclidean distances between the reference and user’s gaze positions during the calibration. The third one is the amount of movement in the user’s gaze position between the previous and current images. Experiments on the accuracy of various gaze tracking methods were conducted using four types of markers and three types of marker display methods during calibration. Through them, the validity of a fuzzy-based estimation system was verified using the feature values and results according to each calibration method. Through the experimental results of this study, the correlation, gradient, and R^2^ were calculated between the real gaze errors and each defuzzification method using the Min or Max rule. From them, the validity of the fuzzy-based gaze error estimation system was proven. In addition, by calculating a *t*-test and Cohen’s d between the defuzzification methods using the Min or Max rule, it was shown that the COG method of the Min rule was the most appropriate.

In the future, a follow-up study will be carried out on the estimation of gaze tracking accuracy based on a neuro-fuzzy system. In addition, the convenience of various calibration methods will be measured through user brainwaves, as well as through a subjective evaluation.
